# Role of stereotactic body radiotherapy in multidisciplinary management of liver metastases in patients with colorectal cancer

**DOI:** 10.1007/s11604-022-01307-9

**Published:** 2022-07-20

**Authors:** Naoko Sanuki, Atsuya Takeda, Yuichiro Tsurugai, Takahisa Eriguchi

**Affiliations:** 1 Department of Radiology, Yokkaichi Municipal Hospital, 2-2-37, Shibata, Yokkaichi, Mie 510-8567 Japan; 2 Radiation Oncology Center, Ofuna Chuo Hospital, 6-2-24, Ofuna, Kamakura, Kanagawa 247-0056 Japan

**Keywords:** Colorectal cancer, Liver metastases, Stereotactic ablative body radiotherapy, Oligometastases, Radiation therapy

## Abstract

In the treatment of colorectal cancer patients with distant metastases, the development of new anticancer agents has considerably prolonged progression-free survival. Such survival benefits attributed to chemotherapy have increased the relative significance of local therapy in patients with limited metastases. The liver is recognized as the most common site of metastasis of colorectal cancer because of the intestinal mesenteric drainage to the portal veins. Hepatic resection of isolated liver metastases of colorectal cancer is the only option for a potential cure. However, hepatic metastases are resectable in only approximately 20% of the patients. For remaining patients with high-risk resectable liver metastases or those who are unfit for surgery, less invasive, local therapies including radiation therapy (stereotactic body radiation therapy, SBRT) may have a potential role in treatment. Although the local control rate of SBRT for colorectal liver metastases has room for improvement, its less-invasive nature and broad indications deserve consideration. Future research should include SBRT dose escalation or the selection of patients who benefit from local ablative therapies. SBRT may offer an alternative, non-invasive approach for the treatment of colorectal liver metastases in a multidisciplinary treatment strategy.

## Introduction

Although cancer metastases are historically regarded as incurable, with the development of treatment modalities, an increasing number of patients with cancer survive in the long term. The oligometastatic hypothesis was first proposed by Weichselbaum and Hellman in 1995 [1]. The term oligometastases indicates an intermediate state between localized and widespread disease. Since then, growing clinical data have supported the importance of classifying cancer metastasis as a spectrum of diseases. Evidence has emerged that patients with oligometastases can be cured by resecting these lesions.

Colorectal cancer is one of the most frequently reported tumor histologies in a surgical series of oligometastases, and the liver is recognized as the most common site of metastasis of colorectal cancer owing to intestinal mesenteric drainage to the portal veins [2]. Managing liver metastasis in these patients is challenging. This article focuses mainly on liver metastasis of colorectal cancer, with a particular focus on curative liver metastasis treatment.

## Treatment strategies for liver metastases of colorectal cancer

### Selection of liver resection candidates with liver metastasis

The development of new systemic agents for the treatment of colorectal cancer has greatly prolonged the survival of colorectal cancer patients with distant metastases [3]. Such prolongation of survival attributed to chemotherapy has increased the relative significance of local therapy for patients with limited metastases. Hepatic resection of isolated liver metastases of colorectal cancer is the only option for a potential cure. Although the principle of treatment for patients with distant metastases is systemic therapy, liver and lung metastases of colorectal cancer had been one of the few conditions for which resection of the metastatic lesion was recommended before the concept of oligometastases became widespread. Current guidelines also support local therapy, mainly aimed at resection, to sites of metastasis of colorectal cancer [4, 5].

Liver metastases of colorectal cancer require multidisciplinary evaluation. A patient with metastatic liver disease first needs to be defined according to whether the disease site is resectable or if it may be resectable after systemic treatment. Hepatic resection is a highly invasive procedure, and it is important to select appropriate patients for the surgery, which depends on several factors, including *oncologic factor, anatomical factor*, and *patient tolerance*.

With regard to *oncologic factor*, several clinicopathologic factors have been shown to be independent predictors of outcomes in patients with resected colorectal liver metastases, and they are combined to form a clinical risk score [6]. In a retrospective study of 612 patients with over 10 years of follow-up who underwent hepatectomy for colorectal liver metastases [7], preoperative clinical risk scores based on five preoperative factors were used: node-positive primary, disease-free interval (< 12 months), number of hepatic metastases (> 1), hepatic metastasis (> 5 cm), and carcinoembryonic antigen (CEA) (> 200 ng/mL). As a result, the 5-year overall survival of the low-risk group defined by those with two or fewer clinical risks was 50%, with a 10-year overall survival rate of 30%. Based on the actual 10-year survivors, they determined the cure rate to be at least 17% and potentially as high as 25%. These data were obtained prior to the introduction of the latest chemotherapeutic agents, such as irinotecan, oxaliplatin, and bevacizumab, and may improve further in the future.

Regarding *anatomical factor*, optimal strategies are attributed to radiological improvements in the diagnostic assessment of colorectal hepatic metastases. Modern computed tomography (CT), magnetic resonance imaging (MRI), and positron emission tomography-CT (PET-CT) techniques enable accurate diagnosis and staging [3]. Patients with resectable metastatic disease and a primary tumor should have both sites resected with curative intent [4, 5]. Postoperative hepatic function can be predicted using CT volumetry. This technique enables the prediction of the remaining volume of hepatic tissue after surgery. Preoperative portal vein embolization and staged liver resection should be considered for hepatic metastatic lesions that are not optimally resectable.

Regarding *patient tolerance* for hepatectomy, adequate liver function, performance status, and other comorbidities were also assessed. Hepatectomy requires to leave at least 20–25% of the total liver volume with adequate inflow, outflow, and biliary drainage [3].

### Patients unfit for hepatectomy

#### Percutaneous ablation

Resection is often contraindicated because of impaired liver function, comorbidities, frailty due to systemic chemotherapy, or intolerance to major hepatectomy. Despite advances in surgical techniques and chemotherapy, only 15–30% of colorectal liver metastases are resectable [3, 8]. For such patients, a less invasive local therapy may be reasonable. Some good options are ablation therapies, such as cryoablation, radiofrequency ablation (RFA), or microwave ablation. These techniques do not interrupt chemotherapy or other concomitant oncological treatments. Other advantages of ablation therapies include less invasiveness than surgery, shorter recovery time, and fewer major complications.

Retrospective analyses of RFA for colorectal liver metastases have shown broad variability in 2-year local control rates (32–76%) and 5-year overall survival rates (14–55%) [9]. The local control rates were lower than with resection, although selection bias was noted. The National Comprehensive Cancer Network (NCCN) guidelines for colon and rectal cancer [4, 5] state that while resection is preferred over local ablative procedures, these local techniques can be considered for liver or lung oligometastases. Ablation therapy is used for non-surgical patients, but are also indicated for small metastases in combination with surgery to treat all visible diseases.

#### Stereotactic body radiation therapy (SBRT)

Historically, radiotherapy with conventional fractionation (i.e., 1.8–2.0 Gy per fraction) has had a limited role in the treatment of liver metastases because of the risk of liver toxicity. In recent years, the concept of ablative radiotherapy, as in radiosurgery for brain tumors, has been applied to the body, and stereotactic body radiation therapy (SBRT) delivers higher doses per fraction (i.e., 10–20 Gy per fraction) to a target volume while sparing unnecessary doses to surrounding normal tissue. SBRT has demonstrated excellent local control compared with conventional fractionation in various small tumors. For example, SBRT results in minimal morbidity and provides high local control rates and is an established treatment option for medically inoperable early-stage lung cancer or for those with advanced age. For localized hepatocellular carcinoma, SBRT has been associated with high local control rates, mostly in the range of 70–90% at 1–2 years [10]. Therefore, SBRT is expected to play a significant role in the treatment of oligometastases.

NCCN guidelines for colon and rectal cancer mention that SBRT is considered for patients with oligometastatic disease [4, 5]. It is also stated that SBRT is a reasonable option for patients in whom resection cannot be performed. As many patients are not surgical candidates and have diseases that cannot be ablated with clear margins, SBRT may be a reasonable option for those who otherwise have to continue systemic chemotherapy for a limited disease burden.

## Overview of SBRT for liver oligometastases

### Treatment results of SBRT

Several studies have investigated the use of SBRT for the treatment of oligometastases. Table 1 shows the outcomes of prospective studies of SBRT for liver metastases, including colorectal cancer. In a prospective study that included approximately half of the patients with colorectal cancer, local control was approximately 80%, which was slightly lower than that for primary lung cancer. In addition, data on overall survival are immature; the 2-year overall survival rates vary from 32–83%, probably owing to patient heterogeneity [11]. A variety of fractionation schedules have been applied, ranging from single fraction to hypofractionation regimens, with the majority of the published studies applying a total of 30–60 Gy in 3–5 fractions.

**Table 1:** Prospective studies of SBRT for liver metastases partially including those from colorectal cancer

Author (year)	Number/lesions	Follow-up duration (months)	Number of metastases	Size, cm	Colorectal cancer (%)	Dose/fractions	Toxicities	Local control	Overall survival
Mendez Romero (2006) [40]	25/34	13	1–3	≤ 7	14%	30–37.5 Gy/3fractions	2%(acute) ≥ grade 3 1%(late) ≥ grade 3	86% (2 year)	62% (2 year)
Rusthoven (2009) [41]	47/63	16	1–3	≤ 6	32%	30–60 Gy/3fractions	< 2%(late) grade3-4	92% (2 year)	30% (2 year)
Lee (2009) [42]	68	11	1–8	Not reported	59%	27.7–60 Gy/6fractions	10% (acute) grade 3–4	71% (1 year)	47% (1.5 year)
Ambrosino (2009) [43]	27	13	1–3	< 6	41%	35–60 Gy/3fractions	No ≥ grade 3	74%	not reported
Rule (2011) [44]	27/36	20	1–5	≤ 7.8 (median 2.5)	44%	30/3fr, 50 Gy/5fractions, 60 Gy/5fractions	No ≥ grade 2	56–89% (2 year)	55% (2 year)
Goodman (2010) [45]	26	17	1–5	< 5	23%	18–30 Gy/1fractions	No ≥ grade 3	77% (1 year)	49% (2 year)
Scorsetti (2013) [46]	61	24	1–3	≤ 6	48%	75 Gy/3fractions	No ≥ grade 3	91% (2 year)	40% (2 year)

### SBRT dose escalation for colorectal liver metastases

There are limited reports on the results of SBRT for liver metastases exclusively aimed at colorectal cancer. Table 2 shows the treatment outcomes, including liver metastases of colorectal cancer. Overall, the local control rates were not as high as those with surgery. Several reports suggest that colorectal liver metastases are more radioresistant; local control appears to be influenced by tumor size and radiation dose [12, 13], supporting the importance of dose escalation for colorectal liver metastases. According to a systematic review evaluating the efficacy of SBRT for colorectal liver oligometastases, the pooled 2-year local control and overall survival rates were 59.3% (95% confidence interval [CI], 37.2–81.5) and 56.5% (95% CI, 36.7–76.2), respectively [14]. Takeda et al. investigated the treatment outcomes and toxicities in patients with oligometastases of colorectal cancer treated with SBRT using risk-adapted, very high-, and convergent-dose regimens. Twenty-one patients (12 and 9 in the liver and lung, respectively) with 28 oligometastases were administered SBRT with a total dose of 50–60 Gy in five fractions prescribed to the 60% isodose line of the maximum dose covering the surface of the planning target volume [15]. The 2-year local control rate was 100% with a median follow-up duration of 28 months. Disease-free and actuarial overall survival rates were 55% and 79%, respectively, with no severe toxicities (≥ grade 3) occurring during follow-up. When only dose-escalated regimens were analyzed in prospective studies, the 2-year local control rate was 81–100%, respectively [3]. Therefore, dose-escalated SBRT may be an alternative to surgical resection of oligometastases in colorectal cancer.

**Table 2:** Prospective studies of SBRT exclusively for colorectal liver metastases

Author (year)	Design	Number/lesions	Follow-up duration (months)	Number of metastases	Size, cm	Dose/fractions	Toxicities	Local control	Overall survival
van der Pool (2010) [47]	Phase II	20/31	26	1–3	< 6.2 ( median 2.3)	37.5-45 Gy/3 fractions	2 grade 3 hepatic toxicity	74 (2 year)	83% (2 year)
Chang (2011) [48]	Prospective	65/102	14	1–4	not reported	22–60 Gy/1–6 fractions	3%(acute) grade 3–414% (late) ≥ grade 3	55% (2 year)	28% (2 year)
Scorsetti (2015) [49]	Phase II	42	24	1–3	≤ 3 cm, 55% > 3 cm, 45%	7 5 Gy/3 fractions	No ≥ grade 3	91% (2 year)	65% (2 year)
McPartlin (2017) [50]	Phase I/II	60	28	1–5	< 15	23–62 Gy/6 fractions	2 acute grade 3 thrombocytopenia	50% (1 year)26% (4 year)	25% (2 year)

The indications for surgery vary depending on the tumor location, number of lesions, age, and complications, and only a select number of patients undergo the procedure. SBRT, on the other hand, does not require such complex decisions and processes, is less invasive, and can often be performed on elderly patients or those with complications. This may provide an opportunity for intensive local treatment in a broader population than the more stringent indications for surgery. If dose-escalated SBRT can achieve results comparable to those of surgery, it may offer a potentially curative alternative to surgery for patients in whom resection cannot be performed. In addition, the reduced invasiveness of local ablative therapies is advantageous in salvage treatment over re-hepatectomy in cases of recurrence.

It is not completely understood why metastases of colorectal cancer are less radiosensitive and have poorer control rates with radiotherapy. In addition to dose and size, chemotherapy rates and genetic abnormalities have also been reported to affect local control rates in SBRT [12, 13]. Some explanations include hypoxic cells in heterogeneous proportions of colorectal metastatic lesions leading to a decrease in radiosensitivity, microscopic extension of oligometastases, or cancer-associated fibroblasts that may compromise local control [16].

### Indication and safety of SBRT

Patients who are SBRT candidates for liver metastases need to have an adequate hepatic function. The liver is a parallel functioning organ that can receive high doses of radiation as long as sufficient normal liver volume is spared. The volume of the uninvolved liver depends on the number and size of lesions. General candidates for liver SBRT have up to three tumors, with the size of each tumor being up to 5–6 cm. Tumors need to be located sufficiently away from the bowels, and typically greater than 700 mL of normal liver tissue must receive less than 15 Gy over 3 fractions [17]. Unlike hepatocellular carcinoma, with cirrhosis in the liver parenchyma, the indications for liver SBRT for liver metastases are broader, and dose escalation is deemed tolerable for such patients.

The safety and effectiveness of SBRT have been evaluated in retrospective and prospective studies of liver metastases with minimal toxicity. Phase I studies starting in the mid-1990s revealed a safe dose escalation of liver SBRT for liver metastases, with most studies treating a limited number of liver metastases [18]. Radiation-induced liver disease after SBRT is rare in patients with liver metastases. Minor complications include loss of appetite, nausea, and low-grade fever. Moderate complications, such as increased liver enzymes and gastrointestinal or severe skin complications, can occur occasionally [12]. Neighboring critical organs, such as the stomach and duodenum, are also potential sites for severe complications. The low incidence of serious complications should be seen as the result of careful indications and treatment planning. In addition, it should be noted that adjacent vital organs such as the stomach and duodenum, as well as hilar vessels such as the bile ducts, are potential sites for serious complications, and this is also disadvantageous for colorectal liver metastases requiring dose escalation.

### Complementary role of SBRT and other local ablative therapies

Ablative local therapies may have the potential to achieve durable effects in oligometastatic liver tumors. The European Organization for Research and Treatment of Cancer (EORTC) 40,004 trial randomized 119 patients with unresectable liver metastasis to receive systemic therapy alone or systemic treatment with RFA. As a result of the randomized phase II trial, overall survival and progression-free survival were improved in the combined modality arm, with 5- and 8-year survival rates of 43% and 36% for the combined modality compared to 30% and 9% for chemotherapy alone. Although the evidence for RFA as a whole for metastatic liver tumors is not necessarily high, it appears, with this randomized comparison data, that RFA is being applied to patients who are not eligible for resection per the guidelines [4, 5]. While SBRT lacks such data, it needs to be shown that SBRT is more beneficial than chemotherapy alone in cases of a localized disease in medically inoperable or high-risk patients.

SBRT and other local ablative therapies, such as RFA, share certain roles in situations where surgery is not possible. They are considered complementary, as is the case with hepatocellular carcinoma. In a comparison of RFA versus SBRT using inverse probability treatment weighting to adjust for potential imbalances in treatment modality, size is a risk factor, and SBRT has a better control rate when the tumor diameter is > 2 cm [19]. Overall, metastases > 3 cm or centrally located lesions close to vascular structures that are not amenable to thermal ablation are good candidates for SBRT.

### Target delineation and its margins in SBRT for colorectal liver metastases

One of the most important aspects of the target definition of SBRT is defining appropriate margins around the tumor. Determining the outline of liver metastases on radiological images is challenging. Focusing on pathologic tumor margins, three types of growth patterns have been described in colorectal liver metastases on gross pathology [20, 21]. First, *the capsulated growth pattern* is characterized by the presence of a fibrous capsule that can be identified on both CT and MR imaging by progressive enhancement from the arterial to delayed phases using extracellular contrast agents because of its fibrous component. Second, the liver cells are progressively displaced by metastatic lesions with *the pushing growth pattern*. Third, with *the infiltrative growth pattern*, tumor cells invade the surrounding hepatic tissue. These patterns are likely to be influenced by cancer-associated fibroblasts around the tumor, also known as desmoplastic reactions, which consist of high-density fibrosis [16, 22]. It is important to recognize the existence of these subtypes and their variability in tumor delineation during SBRT treatment planning.

Most residual tumor cells of colorectal liver metastases after chemotherapy are located in the tumor periphery. Therefore, metastases with a poorer response to chemotherapy have a larger amount of peripherally remaining tumor [23–25]. This is utilized to determine the efficacy of chemotherapy at *the tumor/normal liver interface* (TNI), and the thickness of the tumor cells around TNI has been reported to correlate with prognosis [24, 26]. There are various opinions regarding the margin of resection required for surgery, and some reports indicate that a small margin of ≤ 1 cm or even less does not affect prognosis as long as the margins are negative. In target contouring of SBRT, it is important to keep in mind that there are many tumor cells at the margins of the tumor.

## Combination of chemotherapy and local therapies in colorectal liver metastases

### Potentially reduced liver reserve due to chemotherapy

For patients with a high tumor burden requiring major hepatectomy, preoperative chemotherapy is beneficial, leading to the conversion from unresectable to resectable [6, 7, 27]. Other benefits include size reduction of liver metastases to facilitate complete resection, eradiation of microscopic disease, and preoperative assessment of chemosensitivity and patient tolerance.

One of the considerations for preoperative chemotherapy in the setting of hepatic metastasectomy is the risk of chemotherapy-induced liver injury and the consequent postoperative liver failure. Preoperative chemotherapy can induce parenchymal changes in the liver through steatosis or steatohepatitis [28]. It has been reported that liver injury occurs in 23% of perihepatic pathological findings after chemotherapy plus hepatectomy [29, 30]. In addition, the number of chemotherapy cycles has been reported to be correlated with postoperative complications. Therefore, fewer than 6–7 cycles of chemotherapy and a 4- to 5-week delay between chemotherapy and surgery have been used to reduce the risk of postoperative liver failure. Among the chemotherapies for colorectal cancer, oxaliplatin and irinotecan are known to cause fatty liver disease and hepatic sinusoidal damage. To avoid such hepatotoxicity, the NCCN guidelines recommend that liver metastases treated with preoperative chemotherapy should be resected as soon as they become resectable. In cases of SBRT for colorectal liver metastases as is feasible for SBRT, attention should be paid to liver reserve in patients who have received repeated courses of chemotherapy [4, 5].

### Disappearing liver metastases

In patients receiving conversion chemotherapy, metastatic liver tumors sometimes disappear on preoperative imaging, which is known as disappearing liver metastasis (DLM) [31, 32]. The incidence of DLM varies (7–48%) depending on the heterogeneity in patient characteristics, treatment, or imaging modalities used. DLMs are mostly reported in retrospective studies, and their definitions and imaging modalities vary among studies, although no universal approach exists for the assessment of DLM. DLM, defined as a complete response on imaging, does not necessarily indicate a complete clinical or pathological response. It has been reported that in situ recurrences were observed in 78% of patients with DLM if left unresected, suggesting that an active tumor was still present, although not visible. In fact, local residual disease at the site of the disappearance of metastasis is still found in 11–67% at the time of operation [33–35]. These findings suggest that all lesions present before chemotherapy should be removed, even if they appear as a complete response on imaging after chemotherapy.

Although contrast-enhanced CT is the gold standard for colorectal cancer imaging studies, gadoxetic acid (Gd-EOB-DTPA)-enhanced MR imaging (EOB-MRI) has increased sensitivity, especially for liver metastases [36, 37]. Since decreased sensitivity of CT and PET imaging after preoperative chemotherapy has been reported [32], it is desirable to evaluate liver metastases using MRI prior to SBRT. As small liver metastases are more likely to disappear with preoperative chemotherapy, some investigators have advocated marking these metastases with a fiducial before chemotherapy to facilitate intraoperative localization of possible DLM. Since fiducial markers were originally part of the procedure in liver SBRT to minimize the uncertainty related to internal motion during treatment delivery, the placement of a fiducial marker for SBRT candidates before preoperative chemotherapy can be a reasonable option for such tumors. On the other hand, if SBRT is indicated from the beginning and the intact lesions themselves are considered to be feasible for SBRT, it may be a good idea to treat them before they disappear with preoperative chemotherapy because in resectable cases, chemotherapy is known to improve disease-free survival but has little significance in improving overall survival [38, 39].

## Future perspective

Although SBRT for colorectal liver metastases has achieved promising outcomes with low morbidity, the reported overall survival periods are short. In addition, there are no reports with high-level evidence comparing the efficacy of SBRT with standard therapy. More data are needed to determine whether SBRT can control liver metastases for a long duration and whether it can be as effective as surgery by dose escalation. It is also essential to correlate biomarkers with clinical data to select patients who would benefit from curative local therapies.

## Conclusion

Localized liver metastases of colorectal cancer are expected to be curative if resectable. For those with high-risk or who are unfit for hepatectomy, ablative local therapies, including SBRT, have the potential to achieve durable effects for such diseases with a shorter treatment duration and hospital stay as well as a better quality of life. SBRT can be indicated in a broader range of patients than in surgical candidates, including those with local recurrence after metastasectomy. However, local control of SBRT is yet to be satisfactory, and dose escalation should be attempted to achieve comparative local control over surgery. In addition, optimal target setting based on careful imaging evaluation after chemotherapy is important for better outcomes. SBRT aimed at a cure will improve the overall prognosis of colorectal cancer in the context of a multidisciplinary treatment strategy.
